# Quantitative reproducibility analysis for identifying reproducible targets from high-throughput experiments

**DOI:** 10.1186/s12918-017-0444-y

**Published:** 2017-08-11

**Authors:** Wenfei Zhang, Ying Liu, Mindy Zhang, Cheng Zhu, Yuefeng Lu

**Affiliations:** 1 Department of Biostatistics, Columbia University, New York, NY USA; 2 Sanofi, Framingham, MA USA

**Keywords:** Reproducibility, High-throughput experiment, Bayesian classification, Empirical Bayes, Gaussian mixture, EM algorithm

## Abstract

**Background:**

High-throughput assays are widely used in biological research to select potential targets. One single high-throughput experiment can efficiently study a large number of candidates simultaneously, but is subject to substantial variability. Therefore it is scientifically important to performance quantitative reproducibility analysis to identify reproducible targets with consistent and significant signals across replicate experiments. A few methods exist, but all have limitations.

**Methods:**

In this paper, we propose a new method for identifying reproducible targets. Considering a Bayesian hierarchical model, we show that the test statistics from replicate experiments follow a mixture of multivariate Gaussian distributions, with the one component with zero-mean representing the irreproducible targets.

**Results:**

A target is thus classified as reproducible or irreproducible based on its posterior probability belonging to the reproducible components. We study the performance of our proposed method using simulations and a real data example.

**Conclusion:**

The proposed method is shown to have favorable performance in identifying reproducible targets compared to other methods.

## Background

In biological research, high-throughput assays, such as microarrays, are widely used to effectively select potential targets by studying a large number of candidates in a single experiment. However a high-throughput assay is often subject to substantial variability. Reproducibility of high-throughput assays, such as the level of agreement across replicate samples, test sites or data analytical platforms, is a concerned topic in scientific applications, and has been discussed in [1] for microarray and [2] for ChIP-seq technology. Therefore quantitative analysis for the reproducibility of high-throughput assays is an important exercise for evaluating the reliability and robustness of scientific discoveries across studies.

Reproducibility is nonstandard and unsettled across the sciences. Goodman et al. [3] provides a survey on the papers with the word reproducibility included in titles, abstracts and keywords, and concludes that the interpretation of reproducibility varies among different papers. Goodman et al. [3] further allies the word reproducibility in the papers and classifies them into three terms: methods reproducibility, results reproducibility and inferential reproducibility. In [3], methods reproducibility refers to the provision of enough detail about study procedures and data so the same procedures could, in theory or in actuality, be exactly repeated, such as [1] and [2]; results reproducibility refers to obtaining the same results from the conduct of an independent study whose procedures are as closely matched to the original experiment as possible, such as [4] and [5]; Inferential reproducibility refers to the drawing of qualitatively similar conclusions from either an independent replication of a study or a reanalysis of the original study, such as [1] and [2].

In this paper, our reproducibility analysis aims to identify reproducible targets with consistent and significant signals across replicate studies, which belongs to the category of inferential reproducibility as defined in [3]. Our reproducibility analysis is different from meta-analysis, such as [6] and [7]. Meta-analysis combines the data from multiple studies to gain extra power for identifying targets with signals. The identified targets may not necessarily be significant across all studies.

A few methods have been developed for our reproducibility analysis. Hong et al. [8] proposed a permutation based method through estimating the empirical distribution of the rank product. Benjamini & Heller [9] developed a framework for testing partial conjunction hypothesis that the discovery is true in at least *u* studies out of total *n* studies. Most recently, [10] proposed a copula mixture model for estimating the irreproducible discovery rate across studies.

However all existing methods potentially have limitations. The permutation based method [8] can be computationally expensive when dealing with a large number of candidates. Benjamini & Heller method [9] aims at identifying candidates with reproduced signals in a few but not all the studies, which is a related but generally weaker goal than ours. The special case of Benjamini & Heller method testing whether signals are reproduced in all studies is identical to using the largest *p*-value. The copula mixture [10] method builds the copula mixture using the rank transformation of the original data, which might be less powerful than modeling the original data with a proper probabilistic model as in our proposed method. A major drawback of both Benjamini & Heller method [9] and the copula mixture [10] method is that they both use the significant score of signals, such as *p*-value, without taking into account the directionality of signals, hence is prune to selecting candidates with significant scores but different directions across studies. For example, in the context of two replicate microarray studies with a treatment and a control group, consider genes with significant *p*-values in both experiments, but are up-regulated in one study and down-regulated in the other. Although those genes have inconsistent signals across studies, both methods will likely classify them as reproducible based on *p*-values alone. In contrast, our proposed method models the test statistics directly and is expected to correctly classify those genes as irreproducible most of the time.

In this paper, we propose a Bayesian hierarchical model and show the test statistics from replicate studies can be approximated by a mixture of multivariate Gaussian distributions. The proposed Gaussian mixture model classifies the signals into three components: one irreproducible component and two reproducible components for consistent up-regulated and down-regulated signals respectively. The posterior probability of belonging to the reproducible components is used as a measure for reproducibility.

## Methods

For simplicity, we will introduce our method in the context of microarray studies but it can be generalized to studies of other high-throughput assays. We consider *I* replicate microarray studies for *p* genes. In this paper, we focus on the situation of two replicate studies *I*=2, although our method can be readily extended to the case with more than two studies. We assume a study includes two groups, e.g., the treatment and control group, with sample size equal to *n*_*ik*_ for group *k*, *k*=1,2, in the *i*-th study. Let *x*_*gijk*_ be the normalized and transformed measurement of gene expression of the *j*th sample from group *k* for gene *g* in the *i*-th study. The test statistics of two-sample unpaired t-test for gene *g* in the *i*-th study is

$${}\begin{aligned} d_{gi}&=\frac{\bar x_{gi2}-\bar x_{gi1}}{s_{gi}},\text{where}\\ \bar x_{gi1}&=\sum_{j=1,\cdots,n_{i1}}x_{gij1}/n_{i1},\bar x_{gi2}=\sum_{j=1,\cdots,n_{i2}}x_{gij2}/n_{i2}\\ s_{gi}&=\left[(1/n_{i1}+1/n_{i2})\left\{\sum_{j=1,\cdots,n_{i1}}(x_{gij1}-\bar x_{gi1})^{2}\right.\right.\\ &\left.\left.\quad+\sum_{j=1,\cdots,n_{i2}}(x_{gij2}-\bar x_{gi2})^{2} \right\}/(n_{i1}+n_{i2}-2)\right]^{1/2} \end{aligned} $$

We present an empirical Bayesian hierarchical model to account for various sources of variability. When the sample size *n*_*ik*_ is reasonably large, say *n*_*i*1_+*n*_*i*2_≥30, the test statistics *d*_*gi*_ is well approximated by a normal distribution:

1 $$\begin{array}{@{}rcl@{}} d_{gi} | \mu_{gi} \sim \mathcal{N}(\delta_{S_{i}}\mu_{gi}, 1) \end{array} $$

where *μ*_*gi*_ is the expected group mean difference for gene *g* in the *i*-th study, and $\delta _{S_{i}}=\tilde {\sigma }_{i}^{-1}(1/n_{i1}+1/n_{i2})^{-1/2}$ with $\tilde {\sigma }_{i}$ being the common standard deviation for {*x*_*gij*1_}, *j*=1,2,…,*n*_*i*1_ and {*x*_*gij*2_}, *j*=1,2,…,*n*_*i*2_. When the sample size is small, the same procedure as in [11] can be applied to construct z-tests based on two-sample t-tests. For simplicity we assume the within-group between-sample standard deviation is the same for all the genes. The general case can be derived in a similar fashion but a bit more tedious.

For the expected group mean difference *μ*_*gi*_, we assume it follows

2 $$\begin{array}{@{}rcl@{}} \mu_{gi} | \mu_{g} \sim \mathcal{N}\left(\mu_{g},\sigma_{g}^{2}\right) \end{array} $$

where *μ*_*g*_ is the “true" group mean difference for gene *g* across all studies and $\sigma _{g}^{2}$ models the between-study variability due to various experiment conditions.

Furthermore we assume *μ*_*g*_ is from a mixture distribution

3 $$\begin{array}{@{}rcl@{}} \mu_{g} \sim \pi_{0}I_{\{0\}} + \pi_{1}\mathcal{N}\left(\mu_{G_{1}},\sigma_{G_{1}}^{2}\right) + \pi_{2}\mathcal{N}\left(\mu_{G_{2}},\sigma_{G_{2}}^{2}\right) \end{array} $$

where *π*_*i*_≥0, *i*=0,1,2, with *π*_0_+*π*_1_+*π*_2_=1, $\mu _{G_{1}}>0$ and $\mu _{G_{2}}<0$. The distribution has three components: the null case where there is no differentially expressed gene, the “up-regulated” case where the treatment stimulates the gene expression, and the “down-regulated” case where the treatment suppresses the gene expression. Generally for microarray studies *π*_0_≃1. Similar mixture models have been considered in [11–16]. Particularly we choose to model the cluster of up-regulated (or down-regulated) genes with a Gaussian distribution for the computational convenience, same as in [12]. Alternative choices include the semiparametric mixture model in [11, 14], mixture of Gaussian distributions in [13, 15] and mixture of t-distributions in [16].

We can show that the test statistics (*d*_*g*1_,*d*_*g*2_) follow a Gaussian mixture model. The derivations are standard by repeatedly applying the law of total expectation and the law of total variance and thus omitted. The mixture model is

4 $$ \begin{aligned} (d_{g1},d_{g2}) \sim \pi_{0}\mathcal{N}(\mu_{0},\Sigma_{0})&+\pi_{1}\mathcal{N}(\mathcal{\mu}_{1},\Sigma_{1})\\ &+\pi_{2}\mathcal{N}(\mathcal{\mu}_{2},\Sigma_{2}), \end{aligned}  $$

where $\mathcal {N}(\mathcal {\mu }_{l},\Sigma _{l})$ (*l*=0,1,2) is the biviariate normal distribution with mean vector $\mathcal {\mu }_{l}$ and covariance matrix *Σ*_*l*_. Let *I*_2_ and *J*_2_ be the identity matrix and the square matrix of ones respectively, both with order 2. This mixture model classify the candidates into three components: $\mathcal {N}(\mathcal {\mu }_{0},\Sigma _{0})$ is the irreproducible component with zero-mean $\mathcal {\mu }_{0}=(0,0)^{T}$ and covariance structure $\Sigma _{0}=\left (\sigma _{g}^{2}+1\right)I_{2}$; $\mathcal {N}(\mathcal {\mu }_{1},\Sigma _{1})$ and $\mathcal {N}(\mathcal {\mu }_{2},\Sigma _{2})$ are two reproducible components with $\mathcal {\mu }_{1}=(\delta _{S_{1}}\mu _{G_{1}}, \delta _{S_{2}}\mu _{G_{1}})>0$ and $\Sigma _{1}=\left (\sigma _{g}^{2}+1\right)I_{2} + \sigma _{G_{1}}^{2}J_{2}$ representing the up-regulated genes, and $\mathcal {\mu }_{2}=(\delta _{S_{1}}\mu _{G_{2}}, \delta _{S_{2}}\mu _{G_{2}})<0$ and $\Sigma _{2}=\left (\sigma _{g}^{2}+1\right)I_{2} + \sigma _{G_{2}}^{2}J_{2}$ representing the down-regulated genes, where the inequalities are meant to be interpreted component-wise.

Note with increased sample sizes or decreased within-group between-sample variability, the mean $\mathcal {\mu }_{1}$ and $\mathcal {\mu }_{2}$ of the reproducible components move further away from the origin, making the three components more separable. Also note the test statistics from replicate studies have zero correlations in the irreproducible components; in the reproducible components, the correlations become larger when the between-study variability becomes smaller; for all components, the variance is smaller with less between-study variability, resulting in more separable components.

Under the Gaussian mixture model, the posterior probability of (*d*_*g*1_,*d*_*g*2_) belonging to a component is

5 $$\begin{array}{@{}rcl@{}} p_{gl} = \frac{\pi_{l}\phi(d_{g1},d_{g2}|\mathcal{\mu}_{l},\Sigma_{l})}{\sum_{\ell=0,1,2}\pi_{\ell}\phi(d_{g1},d_{g2}|\mathcal{\mu}_{\ell},\Sigma_{\ell}) },l=0,1,2. \end{array} $$

where *ϕ*(·|·) is the density function of bivariate normal distribution. According to [10], the posterior probability of being in the irreproducible/null component *p*_*i*0_ can be introduced as the individual significant score, namely local false discovery rate. When *p*_*g*0_ is less than a significant level *α*, gene *g* is classified as reproducible.

Next, we consider estimation of the unknown parameters

6 $$ \mathcal{\theta}=(\mu_{1}, \mu_{2}, \Sigma_{0},\Sigma_{1},\Sigma_{2}, \pi_{0},\pi_{1},\pi_{2})  $$

in the mixture model (4) to get the estimate of *p*_*g*0_ for individual genes. It is natural to use the expectation-maximization (EM) algorithm to estimate $\mathcal {\theta }$ by maximizing the log-likelihood of the data [17], i.e.,

7 $$ \begin{aligned} \ell(\mathcal{\theta})&=\sum_{g=1}^{p}\log\{P(d_{g1},d_{g1}|\mathcal{\theta})\}\\ &= \sum_{g=1}^{p}\log\left\{\sum_{l=0}^{2}\pi_{l}\phi(d_{g1},d_{g2}|\mathcal{\mu}_{l},\Sigma_{l})\right\} \end{aligned}  $$

In our algorithm, we start with some initials value for the parameters $\mathcal {\theta }^{0}$, then iterate between two steps: (1) Evaluate the current posterior probabilities *p*_*gl*_ using the current parameters; (2) Maximize the likelihood estimator given current posterior probabilities. The details of the EM procedures are provided in Appendix. Multiple random initial vaues are used to avoid being trapped at the local maximum.

## Simulation studies

In this section, we present numerical simulations to illustrate the performance of our proposed method compared to three existing methods, the copula mixture model [10], Benjamini & Heller method [9], and the rank product method [8]. We use the following model to simulate data

8 $$ \begin{aligned} x_{gijk}&=\mu+ \alpha_{g} + \beta_{i}+(\alpha\beta)_{gi}+ \delta I(k=2)\\ &\quad+\gamma_{g} I(k=2) + (\gamma\beta)_{gi} I(k=2)+\epsilon_{gijk} \end{aligned}  $$

From this model, the mean expression level of gene *g* for group 1 of study *s* is modeled as *μ*_*gs*1_=*μ*+*α*_*g*_+*β*_*i*_+(*α* *β*)_*gi*_, where *μ* is the overall mean; *α*_*g*_ is the main effect of gene *g*; *β*_*i*_ is the main effect of study *i*; (*α* *β*)_*gi*_ is the gene-study interaction. We set *μ*=0, $\alpha _{g} \sim \mathcal {N}(0,1)$, *β*_*i*_=0.1, and $(\alpha \beta)_{gi} \sim \mathcal {N}(0,0.5^{2})$. For non-differentially expressed genes, the mean expression level for both groups are the same, i.e., *μ*_*gs*1_=*μ*_*gs*2_. For differentially expressed genes, (8) models the difference between the two comparison groups as *μ*_*gi*2_−*μ*_*gi*1_=*δ*+*γ*_*g*_+(*γ* *β*)_*gi*_, where *δ* is the fixed effect of group difference; *γ*_*g*_ is the effect of gene on the group difference; (*γ* *β*)_*gi*_ is the gene-study interaction of the group difference. We set *δ*=0, generate *γ*_*g*_ from $\mathcal {N}(2, 0.5^{2})$ or $\mathcal {N}(-2, 0.5^{2})$ to mimic two possible directions of signals, $(\gamma \beta)_{gi}\sim \mathcal {N}(0,0.5^{2})$. *ε*_*gijk*_ is the random error term, and following the distribution $\mathcal {N}(0,0.5^{2})$.

For each simulation run, we generate 2 studies. Each study has two groups with 10 samples per group. We generate *G*=5000 genes per sample and choose the proportions of reproducible genes (*γ*) from (80%, 60%, 40%, 20%, 10%, 5%, 1%). We apply the proposed method and the three existing methods to the simulated data, and classify the genes as reproducible based on two commonly used significant levels (*α*) 0.05 and 0.1. The performance of the four compared methods is evaluated by three criteria, i.e., sensitivity, specificity and misclassification rate. Results from 50 simulations are summarized in Tables 1, 2 and 3 respectively. The results shows our proposed method performs the best among the four methods with the smallest misclassification rates (Table 1), highest sensitivity (Table 2) and highest specificities (Table 3).

**Table 1:** The summary of misclassification rates for the four compared methods under different significant levels (*α*) and proportions of reproducible genes (*γ*)

	The proposed Method		The copula mixture method [10]		Benjamini & Heller method [9]		The rank product method [8]	
	*α*=0.1	*α*=0.05	*α*=0.1	*α*=0.05	*α*=0.1	*α*=0.05	*α*=0.1	*α*=0.05
*γ*=80%	0.007(0.001)	0.008(0.0012)	0.24(0.0708)	0.271(0.0954)	0.025(0.0022)	0.032(0.0025)	0.197(0.0044)	0.25(0.0036)
*γ*=60%	0.007(0.0013)	0.008(0.0013)	0.402(0.0022)	0.404(0.0028)	0.022(0.0017)	0.027(0.002)	0.073(0.0031)	0.099(0.0035)
*γ*=40%	0.005(0.001)	0.006(0.001)	0.568(0.0059)	0.541(0.01)	0.016(0.0017)	0.02(0.0019)	0.02(0.0018)	0.028(0.0021)
*γ*=20%	0.004(8e-04)	0.004(8e-04)	0.166(0.0026)	0.186(0.0015)	0.01(0.0014)	0.013(0.0015)	0.004(9e-04)	0.006(0.0011)
*γ*=10%	0.002(6e-04)	0.002(6e-04)	0.058(0.0104)	0.077(0.0075)	0.007(0.001)	0.008(0.0011)	0.002(5e-04)	0.002(6e-04)
*γ*=5%	0.001(5e-04)	0.001(5e-04)	0.011(0.0038)	0.025(0.0042)	0.004(9e-04)	0.005(0.001)	0.001(4e-04)	0.001(3e-04)
*γ*=1%	0.001(4e-04)	0(4e-04)	0.001(6e-04)	0.002(9e-04)	0.001(7e-04)	0.002(7e-04)	0.001(4e-04)	0(3e-04)

**Table 2:** The summary of sensitivities for the four compared methods under different significant levels (*α*) and proportions of reproducible genes (*γ*)

	The proposed Method		The copula mixture method [10]		Benjamini & Heller method [9]		The rank product method [8]	
	*α*=0.1	*α*=0.05	*α*=0.1	*α*=0.05	*α*=0.1	*α*=0.05	*α*=0.1	*α*=0.05
*γ*=80%	0.992(0.0014)	0.991(0.0016)	0.948(0.0881)	0.905(0.1184)	0.97(0.0027)	0.96(0.0031)	0.754(0.0055)	0.687(0.0045)
*γ*=60%	0.99(0.002)	0.988(0.0021)	0.978(0.0071)	0.956(0.0119)	0.966(0.0028)	0.955(0.0033)	0.878(0.0052)	0.836(0.0058)
*γ*=40%	0.989(0.0024)	0.987(0.0024)	0.975(0.0069)	0.937(0.0161)	0.962(0.0046)	0.951(0.005)	0.949(0.0045)	0.931(0.0051)
*γ*=20%	0.985(0.0037)	0.983(0.004)	0.176(0.0149)	0.069(0.0081)	0.949(0.007)	0.937(0.0076)	0.978(0.0046)	0.972(0.0053)
*γ*=10%	0.984(0.0048)	0.982(0.0051)	0.421(0.1033)	0.228(0.0746)	0.934(0.0098)	0.92(0.0108)	0.985(0.0053)	0.982(0.0055)
*γ*=5%	0.984(0.0069)	0.983(0.0075)	0.773(0.0741)	0.509(0.0832)	0.925(0.0191)	0.909(0.0195)	0.99(0.0049)	0.988(0.0057)
*γ*=1%	0.986(0.0176)	0.984(0.0177)	0.907(0.0592)	0.842(0.0882)	0.866(0.0673)	0.844(0.0706)	0.99(0.0163)	0.99(0.0163)

**Table 3:** The summary of specificities for the four compared methods under different significant levels (*α*) and proportions of reproducible genes (*γ*)

	The proposed Method		The copula mixture method [10]		Benjamini & Heller method [9]		The rank product method [8]	
	*α*=0.1	*α*=0.05	*α*=0.1	*α*=0.05	*α*=0.1	*α*=0.05	*α*=0.1	*α*=0.05
*γ*=80%	0.996(0.002)	0.997(0.0017)	0.009(0.0058)	0.025(0.0152)	0.994(0.0021)	0.999(0.001)	1(0)	1(0)
*γ*=60%	0.998(9e-04)	0.999(7e-04)	0.029(0.0075)	0.057(0.0144)	0.997(0.0015)	0.999(7e-04)	1(0)	1(0)
*γ*=40%	0.999(7e-04)	0.999(6e-04)	0.07(0.0136)	0.139(0.0268)	0.999(7e-04)	1(4e-04)	1(0)	1(0)
*γ*=20%	0.999(4e-04)	0.999(3e-04)	0.999(9e-04)	1(4e-04)	1(3e-04)	1(1e-04)	1(0)	1(0)
*γ*=10%	0.999(4e-04)	1(3e-04)	1(1e-04)	1(1e-04)	1(1e-04)	1(1e-04)	1(2e-04)	1(1e-04)
*γ*=5%	1(3e-04)	1(3e-04)	1(1e-04)	1(0)	1(1e-04)	1(0)	1(3e-04)	1(1e-04)
*γ*=1%	1(3e-04)	1(3e-04)	1(1e-04)	1(0)	1(0)	1(0)	1(4e-04)	1(2e-04)

## Results

In this section, we illustrate our proposed method using a real example. This example includes two microarray studies [18] and [19] comparing idiopathic pulmonary fibrosis (IPF) samples with healthy control samples. Data from both studies are obtained from Gene Expression Omnibus [20]. GSE 28042 [18] measures profiles of peripheral blood mononuclear cell (PBMC) for 75 IPF samples and 16 control samples through GeneChip Human 1.0 exon ST arrays, and GSE 33566 [19] measures profiles of peripheral blood RNA for 93 IPF patients and 30 control samples through Agilent Whole Human Genome Oligonucleotide Microarrays. We only consider the overlap 17708 common genes for reproducibility analysis.

**Fig. 1:**
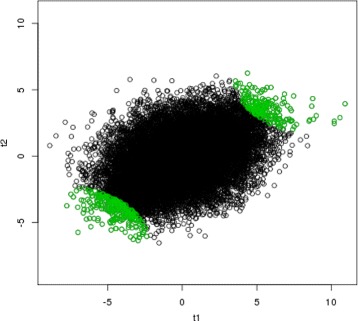
Bivariate plot of test statistics from two studies. The *x* axis represents the test statistics from GSE 28042 study [18], and the *y* axis represents the test statistics from GSE 33566 [19]. The *green* points are the top 500 reproducible genes selected by the proposed method

**Fig. 2:**
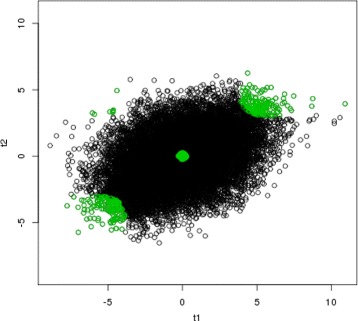
Bivariate plot of test statistics from two studies. The *x* axis represents the test statistics from GSE 28042 study [18], and the *y* axis represents the test statistics from GSE 33566 [19]. The *green* points are the top 500 reproducible genes selected by the copula mixture model [10]

**Fig. 3:**
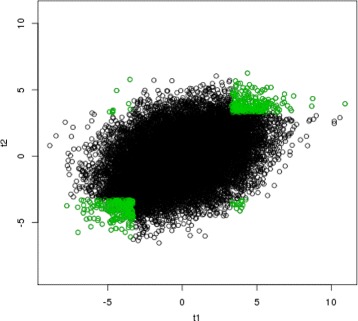
Bivariate plot of test statistics from two studies. The *x* axis represents the t-statistics from GSE 28042 study [18], and the *y* axis represents t-statistics from GSE 33566 [19]. The *green* points are the top 500 reproducible genes selected by Benjamini & Heller method [9]

We apply our proposed method, the copula mixture model [10] and Benjamini & Heller method [9]. The rank product method [8] is too computationally intensive to be applied to this example and thus excluded from this study. Figures 1, 2 and 3 show the results of selected reproducible genes from the three compared methods respectively (green). In all three figures, the *x* axis represents the test statistics from GSE 28042 [18], and the y axis represents the test statistics from GSE 33566 [19]. The top 500 reproducible genes selected by three methods are highlighted in green. As shown in Fig. 1, our proposed method only selects genes with consistently significant signals in both studies. Benjamini & Heller method [9] incorrectly identifies 23 genes (the upper left and bottom right corners of Fig. 2) as reproducible, which actually have opposite directions in two studies. The complete list of the 23 genes incorrectly selected by Benjamini & Heller method [9] is provided in Table 4. The copula mixture model [10] selects 7 genes (Table 5) with opposite directions of signals. It’s also noted that the copula mixture model [10] appears to be less powerful in separating the irreproducible and reproducible genes and has incorrectly selected some insignificant genes (see the center of Fig. 3), likely resulting from the rank transformation. Overall, our method performs favorably in identifying reproducible genes.

**Table 4:** The list of 23 selected genes, which are in the list of the top 500 reproducible genes selected by Benjamini & Heller method [9], but have opposite signs of signals in two studies

	Genes	t-statistics in GSE 28042 [18]	t-statistics in GSE 33566 [19]
1	A1BG	3.34	-3.63
2	ANKRD39	3.93	-3.35
3	CA4	-4.4	4.94
4	CDK14	-4.88	3.34
5	CHCHD2	3.5	-3.65
6	CXCR2	-4.67	3.38
7	HCG27	-4.68	3.29
8	KAT6A	-3.48	3.54
9	MFSD3	4.25	-3.29
10	MMP9	-3.51	5.77
11	MRPL14	4.06	-3.69
12	MRPL15	3.99	-3.38
13	MRPL55	3.63	-3.95
14	NDUFB7	3.79	-3.54
15	NDUFS3	3.98	-3.89
16	PRPS1	3.87	-4.13
17	RBBP6	3.66	-3.67
18	ROMO1	3.33	-3.41
19	SEPHS1	4	-3.44
20	TANC2	-3.59	3.95
21	TCN1	-4.69	3.36
22	TMEM141	3.45	-3.64
23	TRIM33	-4.64	3.47

**Table 5:** The list of 7 selected genes, which are in the list of the top 500 reproducbile genes selected by the copula mixture model [10], but have opposite signs of signals in two studies

	Gene	t-statistics in GSE 28042 [18]	t-statistics in GSE 33566 [19]
1	CA4	-4.4	4.94
2	CDK14	-4.88	3.34
3	CXCR2	-4.67	3.38
4	HCG27	-4.68	3.29
5	MME	-6.05	3.25
6	TCN1	-4.69	3.36
7	TRIM33	-4.64	3.47

## Conclusion and discussion

This paper proposes a new method for identifying consistent and significant signals across replicate high-throughput experiments. Existing methods ignore the directionality of signals, and can incorrectly identify signals with opposite directions as reproducible ones. Our proposed method considers both the significant scores and directions of signals by modeling the test statistics directly, leading to improved performance in selecting reproducible candidates. When the proposed method is applied to a real data example for identifying reproducible genes in studies of idiopathic pulmonary fibrosis samples, it is shown to have better performance in detecting significant and reproducible genes compared to other methods. Simulations also demonstrate that our method compares favorably to the existing methods.

## Appendix

### Expectation-maximization (EM) algorithm to estimate model parameters

The algorithm for estimating $\mathcal {\theta }$ in (6) is an iterative algorithm between Expectation steps and maximization step. We use $\widehat {\mathcal {\theta }}^{v}$ to denote the estimate at *v*th iteration. The algorithm includes the following steps:

- **Step 1: Initial Values** Generate the initial values for $\mathcal {\theta }$ and denote it as $\widehat {\mathcal {\theta }}^{0}$
- **Step 2: Expectation-Step** Continue from the *v*th iteration step with the estimate $\widehat {\mathcal {\theta }}^{v}$. We can obtain the estimated posterior probability $\widehat {p_{gl}}^{v}$ of (*d*_*g*1_,*d*_*g*2_) from (5) by
  9 $$\begin{array}{@{}rcl@{}} \widehat{p_{gl}}^{v}&=&\frac{\widehat{\pi_{l}}^{v}\phi\left(d_{g1},d_{g2}|\widehat{\mathcal{\mu}_{l}}^{v},\widehat{\Sigma_{l}}^{v}\right)}{\sum_{\ell=0,1,2}\widehat{\pi_{\ell}}^{v}\phi\left(d_{g1},d_{g2}|\widehat{\mathcal{\mu}_{\ell}}^{v},\widehat{\Sigma_{\ell}}^{v}\right)},l=0,1,2. \end{array} $$
- **Step 3: Maximization-Step** Update the parameter $\widehat {\mathcal {\theta }}^{v+1}$ by maximizing the log-likelihood function $\ell (\mathcal {\theta })$ in (7) given the current estimated posterior probability $\widehat {p_{gl}}^{v}$. The estimated parameters from the maximization are
  $$\begin{aligned} \widehat{\pi_{l}}^{v+1}&=\sum_{g=1}^{p}\widehat{p_{gl}}^{v}/p,l=0,1,2.\\ \widehat{\mu_{1}}^{v+1}&=\left(\widehat{\mu_{11}}^{v+1},\widehat{\mu_{12}}^{v+1}\right)= \left(\frac{\sum_{g=1}^{p} \widehat{p_{g2}}^{v} d_{g1}}{\sum_{g=1}^{p} \widehat{p_{g2}}^{v}}, \frac{\sum_{g=1}^{p} \widehat{p_{g2}}^{v} d_{g2}}{\sum_{g=1}^{p} \widehat{p_{g2}}^{v}}\right)\\ \widehat{\mu_{2}}^{v+1}&=\left(\widehat{\mu_{21}}^{v+1},\widehat{\mu_{22}}^{v+1}\right)= \left(\frac{\sum_{g=1}^{p} \widehat{p_{g3}}^{v} d_{g1}}{\sum_{g=1}^{p} \widehat{p_{g3}}^{v}}, \frac{\sum_{g=1}^{p} \widehat{p_{g3}}^{v} d_{g2}}{\sum_{g=1}^{p} \widehat{p_{g3}}^{v}}\right) \\ \widehat{\sigma_{g}^{2}}^{v+1}&= \frac{\sum_{g=1}^{p} \widehat{p_{g1}}^{v}\left(d_{g1}^{2}+d_{g2}^{2}\right)}{2\sum_{g=1}^{p} \widehat{p_{g1}}^{v}} -1 \\ \widehat{\sigma_{G_{1}}^{2}}^{v+1}&= \frac{\sum_{g=1}^{p} \left[\widehat{p_{g2}}^{v} \left(d_{g1}-\widehat{\mu_{11}}^{v+1}\right)^{2}+\widehat{p_{g2}}^{v} \left(d_{g2}-\widehat{\mu_{12}}^{v+1}\right)^{2}\right]}{2\sum_{g=1}^{p} \widehat{p_{g2}}^{v}} \\ &\quad-\frac{\sum_{g=1}^{p} \widehat{p_{g1}}^{v}\left(d_{g1}^{2}+d_{g2}^{2}\right)}{2\sum_{g=1}^{p} \widehat{p_{g1}}^{v}} \\ \widehat{\sigma_{G_{2}}^{2}}^{v+1}&= \frac{\sum_{g=1}^{p} \left[\widehat{p_{g3}}^{v} \left(d_{g1}-\widehat{\mu_{21}}^{v+1}\right)^{2}+\widehat{p_{g3}}^{v} \left(d_{g2}-\widehat{\mu_{22}}^{v+1}\right)^{2}\right]}{2\sum_{g=1}^{p} \widehat{p_{g3}}^{v}} \\ &\quad-\frac{\sum_{g=1}^{p} \widehat{p_{g1}}^{v}\left(d_{g1}^{2}+d_{g2}^{2}\right)}{2\sum_{g=1}^{p} \widehat{p_{g1}}^{v}} \end{aligned} $$
- **Step 4: Solution** The algorithm continues between Expectation-Step and Maximization-Step until the following two conditions are satisfied.
  1. The difference between $\widehat {\mathcal {\theta }}^{v} $ and $\widehat {\mathcal {\theta }}^{v+1}$ is less than a small value *δ*_1_ for all their elements;
  2. The change in log-likelihood function $\ell (\mathcal {\theta })$ between two consecutive iterations does not exceed a small value *δ*_2_.
